# Harnessing the Therapeutic Potential of Th17 Cells

**DOI:** 10.1155/2015/205156

**Published:** 2015-05-26

**Authors:** Jonas Bystrom, Taher E. Taher, M. Sherwan Muhyaddin, Felix I. Clanchy, Pamela Mangat, Ali S. Jawad, Richard O. Williams, Rizgar A. Mageed

**Affiliations:** ^1^Bone and Joint Research Unit, William Harvey Research Institute, Queen Mary University of London, London EC1M 6BQ, UK; ^2^College of Pharmacy, Hawler Medical University, Erbil, Iraq; ^3^Kennedy Institute of Rheumatology, University of Oxford, Oxford OX3 7FY, UK; ^4^Department of Rheumatology, Royal Free Hospital, London NW3 2GQ, UK; ^5^Department of Rheumatology, The Royal London Hospital, London E1 4DG, UK

## Abstract

Th17 cells provide protective immunity to infections by fungi and extracellular bacteria as well as cancer but are also involved in chronic inflammation. The cells were first identified by their ability to produce interleukin 17A (IL-17A) and, subsequently, associated with chronic inflammation and autoimmunity. Th17 cells have some gene profile similarity with stem cells and can remain dormant in mucosal tissues for long periods. Indeed, recent studies suggest that functionally distinct subsets of pro- and anti-inflammatory Th17 cells can interchange phenotype and functions. For development, Th17 cells require activation of the transcription factors STAT3 and ROR*γ*t while RUNX1, c-Maf, and Aiolos are involved in changes of phenotype/functions. Attempts to harness Th17 cells against pathogens and cancer using vaccination strategies are being explored. The cells gain protective abilities when induced to produce interferon *γ* (IFN*γ*). In addition, treatment with antibodies to IL-17 is effective in treating patients with psoriasis, psoriatic arthritis, and refectory rheumatoid arthritis. Moreover, since ROR*γ*t is a nuclear receptor, it is likely to be a potential future drug target for modulating Th17 functions. This review explores pathways through which Th17 subsets are induced, the molecular basis of their plasticity, and potential therapeutic strategies for their modulation in diseases.

## 1. Introduction

Different subsets of helper T cells (Th) have been identified based, primarily, on the pattern of cytokines they produce. The Th1 subset is induced to differentiate in response to intracellular pathogens and viruses to produce IFN*γ* and TNF*α* and initiate cellular immunity. The Th2 subset, in contrast, produces interleukin 4 (IL-4), IL-5, and IL-13 and mediates immunity to helminths and parasites as well as initiating humoral immunity. Regulatory T cells, which were discovered subsequently, dampen inflammatory responses against foreign and self-antigens through cell-cell interactions and produce IL-10 and TGF*β* [1]. The most recent addition to effector Th subsets is Th17 cells that were identified in 2006 based on their ability to produce IL-17A [2]. Although the latest to be discovered, subsequent evolutionary studies have established that the Th17 subset is the most ancient one. Hence, immune cells equipped with a nascent T cell receptor (TCR) from the primitive fish lamprey, whose lineage diverged from that of humans 500 million years ago, produce IL-17 but none of the cytokines associated with the other T cell lineages [3]. In mammals, at homeostasis commensal bacteria in the gut induce IL-1*β* production to maintain a basal level of Th17 cells in the lamina propria [4]. However, in response to pathogenic extracellular bacterial and fungal infections at mucocutaneous surfaces in the intestine, the respiratory tract, and the skin, large numbers of naive Th cells differentiate to Th17 cells under the influence of IL-1*β*, IL-6, IL-23, and/or TGF*β* [5]. In addition to producing IL-17A, Th17 cells can produce IL-17F, IL-21, IL-22, IFN*γ*, and GM-CSF [6, 7]. IL-17A, referred to as IL-17 in this review, has pleiotropic properties after binding IL-17 receptors on haematopoietic and nonhaematopoietic cells such as epithelial and endothelial cells [8]. The binding of IL-17 to its receptors triggers intracellular signalling that induces the production of proinflammatory cytokines such as IL-6, C-X-C chemokines such as chemokine 8 (CXCL8), CXCL9, CXCL10, and CXCL11, and beta-defensin-2 [9–11]. During acute infections, Th17 cells recruit neutrophils and, thereby, mediate initial protection from pathogens [8]. Furthermore, IL-21 and IL-22 produced by Th17 cells protect mucosal membranes by inducing the production of antimicrobial proteins, RegIII*β*, and RegIII*γ* and by stimulating B cells [6, 7]. IL-17 is, by itself, a weak activator of other immune cells and studies have shown that the presence of other cytokines, such as TNF*α* or IL-1*β*, is required for maximum effects of the cytokine [12, 13].

After their activation, effector and memory Th17 cells can remain dormant in the mucosa for extended periods of time [14]. A number of recent studies have revealed that Th17 cells show a great degree of functional and phenotypic plasticity. Thus, there is evidence that Th17 cells can change to Th1-like cells or acquire the ability to produce IL-10 which can be beneficial during certain types of infections. For example,* Candida albicans* induces IFN*γ* production by Th17 cells while* Staphylococcus aureus* induces IL-10 [15]. With regard to phenotype, all Th17 cells express CCR6 and most also express CD161 [16]. Th17 cells that only produce IL-17 express CCR4 while IFN*γ*-producing Th17 cells express CXCR3 [17]. In addition to providing protective immunity and driving chronic inflammation, Th17 cells have been suggested to play a dual role in tumour development. Thus, Th17 cells have been implicated in promoting tumour through producing angiogenic factors but, paradoxically, also shown to counteract tumour development by producing IL-17 and IFN*γ* [18]. Treatment of patients with the epidermal skin disorder psoriasis with antibodies to IL-17 or with its soluble recombinant receptor leads to remission [19, 20]. Furthermore, patients with rheumatoid arthritis (RA), psoriatic arthritis, and ankylosing spondylitis have been reported to benefit from treatment with biologic inhibitors of IL-17 [21–23]. However, treatment of patients with Crohn's disease with inhibitors of IL-17 worsens disease, perhaps, highlighting some protective functions for Th17 cells in the gastrointestinal tract [24]. Interestingly, there is some evidence to indicate that the ability of Th17 cells to promote pathology in autoimmune diseases is acquired when the cells gain the ability to produce IFN*γ*. In animal models of disease, these cells were shown to express receptors for either IL-23 or IL-1*β* [25, 26].

This review will explore potential strategies to harness the use of Th17 cells for therapeutic purposes. First, we will review available evidence on the signals that promote the development of Th17 cells and mechanisms that underpin changes to their phenotype. These involve TCR- and cytokine-mediated signals, transcription factors, and epigenetic modifications. Second, studies aimed at employing Th17 cells for vaccination against various organisms and for protection from cancers will be reviewed. We will also discuss advantages and pitfalls of reported experimental strategies and contemplate whether it would be beneficial to alter the phenotype of Th17 cells in human diseases.

## 2. Th17 Cell Development, Transcriptional Regulation, and Functional Plasticity

The available evidence indicates that Th17 cell progenitors, identified by CD161 expression, are present at mucocutaneous sites and in peripheral and cord blood [16]. These cells are induced to differentiate into effector Th17 cells by cytokines that activate a highly regulated transcriptional network involving at least five transcription factors and through epigenetic modifications. Cytokines IL-1*β*, IL-6, TGF*β*, and IL-23 and the lipid mediator PGE_2_ have all been variably reported to be involved in Th17 cell differentiation [27]. An initial medium level of T cell receptor (TCR) engagement activates the nuclear factor kappa B (NF*κ*B) which, in turn, activates the interferon regulatory factor 4 transcription factor (IRF4). In contrast, high level TCR engagement preferentially promotes Th1 cell differentiation. The two transcription factors together with another transcription factor called basic leucine zipper transcription factor ATF-like (BATF), binds multiple sites throughout the chromatin [28–30]. IL-1*β* increases the expression of IRF4 [31] while IL-6 and IL-23 induce the phosphorylation of signal transducer and activator of transcription 3 (STAT3). This leads to the disassociation of STAT3 from the receptor-bound Janus kinase 2 (JAK2). Phosphorylated STAT3 then transmigrates to the nucleus and populates many DNase sensitive chromosomal sites, made accessible by TGF*β*, and stabilizes some of the BATF/IRF4 interactions [28]. IL-23 also induces the expression of a fourth transcription factor, runt-related transcription factor 1 (RUNX1) [32]. RUNX1/3 promotes Th17 differentiation by enhancing expression of the transcription factor ROR*γ*t and increasing its stability at the* Il17* locus [33]. The transcription factor ROR*γ*t is a signature transcription factor for Th17 cells as it binds a number of specific DNA loci critical for the differentiation of the cells [28].

**Figure 1:**
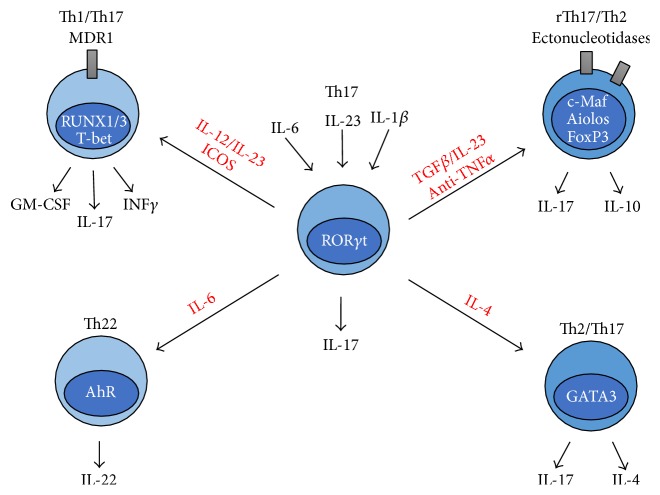
Th17 cell plasticity. Cytokines IL-1*β*, IL-6, and IL-23 activate the Th17 cell-specific differentiation program (centre) through activating the transcription factor ROR*γ*t. IL-12 and IL-23 induce the transcription factors RUNX1/3 and T-bet in Th17 cells leading to IFN*γ* and in some cases GM-CSF production. This is augmented by stimulation of the coreceptor ICOS (upper left). Exposure of Th17 cells to IL-6 can induce IL-22 production (lower left). Treatment with biologic anti-TNF*α* agents or exposure to TGF*β* has been shown to promote IL-10 production accompanied by, in some studies, the expression of the transcription factor FoxP3. Th17 cells can also gain the ability to express ectonucleotidase in response to TGF*β* (upper right). Finally, IL-4 can promote the generation of a Th17/Th2 cell type capable of producing IL-4. These cells express the transcription factor GATA-3 (lower right).

Numerous studies have highlighted the plasticity of Th17 cells [32, 34–36]. Although key Th17-specific loci (e.g.,* Il17a*,* Il17f*, and* Rorc*) are known to be accessible in all Th17 cells, they have also been reported to be easily repressed. TGF*β* has been shown to stabilize the open state of these loci but in its absence both IL-23 and IL-12 suppress IL-17 production while instead enhancing IFN*γ* production in a STAT4- and T-box transcription factor- (T-bet-) dependent manner [34]. Furthermore, the* Ifng* locus was shown to be semiactivated in Th17 cells and to rapidly acquire an additional permissive state in response to IL-12 [35]. IL-12 induces T-bet expression and repressive histone marks in the* Rorc* locus [35]. T-bet then interacts with RUNX1 to disrupt RUNX1/ROR*γ*t interaction and activity [36]. In the presence of IL-12, RUNX1 was also reported to bind to the* Ifng* promoter [32]. T-bet and RUNX1/3 activation is required for maximal IFN*γ* production in “ex-Th17” cells. At low RUNX1 levels, however, and in the presence of Th17-promoting cytokines, the Th17 cell phenotype is retained. Depending on the level of RUNX1 activation and which cytokines are present, the formation of the RUNX1/T-bet complex in Th17 cells leads to the development of IFN*γ* ^+^IL-17^+^ T cells independent of ROR*γ*t expression [32]. IFN*γ* ^+^ Th17 cells have been shown to have the ATP-dependent membrane efflux pump P-glycoprotein/multidrug resistance type 1 (MDR1) [17] (Figure 1).

Dendritic cell- (DC-) induced Th17 cell differentiation in response to* S. aureus* has been shown to induce IL-10 production in addition to IL-17 [15, 37]. Similarly, treatment of RA patients with biologic anti-TNF*α* agents induces IL-10-producing Th17 cells [37]. The ability of Th17 cells to produce IL-10 was shown to be regulated by one of the transcription factors c-Maf and Aiolos [37, 38]. c-Maf, which is also associated with a Th2 phenotype, represses expression of* Rora*,* Runx1*,* Il1r1*,* Ccr6*, and* Tnf *genes [28]. Other studies have shown that the Th17 cells can gain the ability to produce IL-22 and IL-4 with IL-6 shown to induce the production of IL-22 [39, 40]. Th17 cells can also express the transcription factor associated with regulatory T cells [41] (Figure 1). These observations are further evidence to indicate that Th17 cells have the capacity for phenotypic and functional plasticity. In this respect, studies of enriched memory Th17 cells showed that the cells expressed *β*-catenin which is associated with stem cell character and its T cell-specific binding partner T cell factor 1 (Tcf1, also known as Tcf7) [18]. In contrast, expression of Tcf1 is suppressed by T-bet in Th1 cells [18]. In addition, high levels of cyclins and reduced levels of cyclin-dependent kinase (CDK) repressors were reported in Th17 cells [14]. Reduction in CDK repressors has been shown to be essential for self-renewal of haematopoietic stem cells [42]. The chromosomal availability for binding sites for Tcf1 was greatly increased in T cells engineered with constituently active *β*-catenin. In these cells, Tcf1 was found bound to the* Rorc* promoter activating the Th17 cell differentiation [43]. These observations provide insights into how Th17 cells can remain dormant until being stimulated by appropriate antigens. Unlike other T cell subsets, the stem-cell characteristics of Th17 cells apparently endow them with the ability to retain the potential for renewal and functional plasticity for long periods of time. Subsequent to TCR engagement, the cytokine milieu regulates the phenotype of “differentiated” Th17 cells. This ability can be used to harness the therapeutic potential of these cells when considering new vaccine strategies for inducing protective immunity to extracellular pathogens and fungi and for treating patients with cancer and autoimmune diseases.

## 3. Vaccination to Induce Pathogen-Specific Th17 Responses

Th17 cells have a well-described role in immunity against fungi and extra cellular bacteria, such as* Candida albicans* and* Streptococcus pneumoniae*. Infection with these pathogens is generally prevalent in immune compromised individuals, especially in STAT3- and Th17-deficient patients [44]. It was, therefore, proposed that Th17 cell responses can be harnessed by novel vaccine strategies to induce such cells to provide protective immunity against these organisms. An interesting issue as to whether the plasticity of Th17 cells can be exploited for the development of more effective vaccines has been considered. For example, a number of studies have shown that the advantage of a vaccine that relies on inducing Th17 cell-dependent responses would be that the protective immunity, unlike the B cell-mediated immunity, will be independent of pathogen serotype [45]. A further possible advantage of Th17-inducing vaccines would be that infants and immune compromised individuals that do not develop a good antibody response will benefit from long-lived memory Th17 cells [18, 45, 46]. To enhance Th17 responses by vaccination, the use of various adjuvants has been assessed. The bacterial components, muramyl dipeptide (MDP), lipopolysaccharide (LPS), and CpG, augmented Th17 responses [45, 47, 48].

Strategies to develop vaccines that specifically induce Th17 cells in immune compromised individuals have also been actively considered. These efforts were based on key observations regarding the role of Th17 immunity at sites most susceptible to infections in immune compromised individuals. Thus, Th17-mediated immunity to* C. albicans* is important for infections of the upper respiratory tract and the skin [44, 49]. During such infections, fungal antigens activate Dectin-1 and toll-like receptor 2 (TLR2) on dendritic cells and this leads to the production of IL-23 and IL-1*β* [50]. Th17 cells generated in response to* C. albicans*, in turn, induce the production of IFN*γ* and, thus, further augment cellular immunity [15]. A vaccine consisting of a recombinant virulence factor used by* Candida* which has a similar shape to a virulence factor in* S. aureus* (N-terminus of Als3p) with aluminium hydroxide as adjuvant induced protective immunity dominated by Th17 cells that produced both IL-17 and IFN*γ* and recruited neutrophils [51].

In addition to fungal infections in which Th17 cell-mediated immunity plays a critical protective role, these cells are also important in immunity to the gram-positive bacterium,* Streptococcus pneumoniae*. This bacterium causes life-threatening infections of the respiratory tract in immune compromised individuals and, in addition, leads to systemic infection and septic arthritis [52]. There are over 90 different serotypes of* S. pneumonia* and antibiotic resistance can easily develop [53]. In children, natural protection against* S. pneumoniae* is dependent on Th17 cells and this immunity develops before antibody-mediated immunity [46]. During the course of an infection, activated monocytes first recruit Th17 cells which, in turn, recruit neutrophils to kill the bacteria [54–56]. Furthermore, intranasal vaccination with common cell wall polysaccharides, which bind MHCII, has been shown to induce a Th17-dependent, antibody-independent protective immunity [57, 58].

The gram-negative* Pseudomonas aeruginosa* is another pathogen known to induce a Th17 response. This pathogen can cause similar infections as* C. albicans* and* S. pneumonia*, that is, sepsis and respiratory and gastrointestinal tract infections, especially in immune compromised individuals [59]. IL-17 production was noted in response to intranasal vaccination with live attenuated* P. aeruginosa*, or a library of* P. aeruginosa* proteins. The resulting immunity which was mostly dependent on rapid neutrophil recruitment conferred protection to several strains of the bacterium [59, 60].

The role of Th17-mediated protective immunity to the respiratory tract is further highlighted by studies showing immunity to conserved outer membrane proteins from several serotypes of the gram-negative bacterium,* Klebsiella pneumonia* [45]. Vaccination with this bacterium with LPS used as adjuvant induced an MHCII-dependent Th17 cell-mediated immunity [45]. The resulting immune response was serotype-independent and specific to conserved outer membrane proteins. The response was also antibody-independent and lasted for at least four weeks. In contrast, serotype-specific immunity to polysaccharide capsular antigens from* K. pneumonia* induced a transient B cell response [45].

Protection from* Mycobacterium tuberculosis* is also known to involve Th17-mediated immunity. Thus, a study noted that vaccination with* M. tuberculosis* induced protection associated with an IL-17-mediated response [11]. In this study, IL-23 was shown to be essential for the accelerated immune response to prevent bacterial growth and the induction of Th17 cells in the lung. The recall Th17 response occurred concurrently with the expression of chemokines CXCL9, CXCL10, and CXCL11 that, in turn, recruited other CD4^+^ cells that produced IFN*γ* in the lung [11].

Another study reported that immunization with the gram-negative* Bordetella pertussis*, which causes whooping cough, induced immunity that was associated with the induction of Th17 cells. Thus, intraperitoneal immunization with a whole* B. pertussis* vaccine twice, four weeks apart followed by challenge with aerosol inoculation two weeks later, induced a toll-like receptor 4- (TLR4-) dependent production of IL-23 from DCs [61]. This augmented Th1 and Th17 responses and led to protective cellular immunity that involved bacterial killing by activated macrophages.

Although often associated with a Th1 immune response, immunity to the respiratory syncytial virus (RSV) has also been shown to induce a Th17 response in the respiratory tract. A protective Th17-dependent response developed in a mouse model concomitant with allergic asthma and also in infected infants [62, 63]. Interestingly, a* Pertussis* vaccine induced protection from RSV infections in neonatal mice when given intranasally [64]. This vaccination led to modification of the immune response by enhancing mucosal resistance to RSV infection during adulthood. In this setting, IL-17 was produced by Th17 and NK cells and led to the recruitment of neutrophils. In addition, IL-17^+^IFN*γ* ^+^ T cells were shown to contribute significantly to the protection [64].

**Figure 2:**
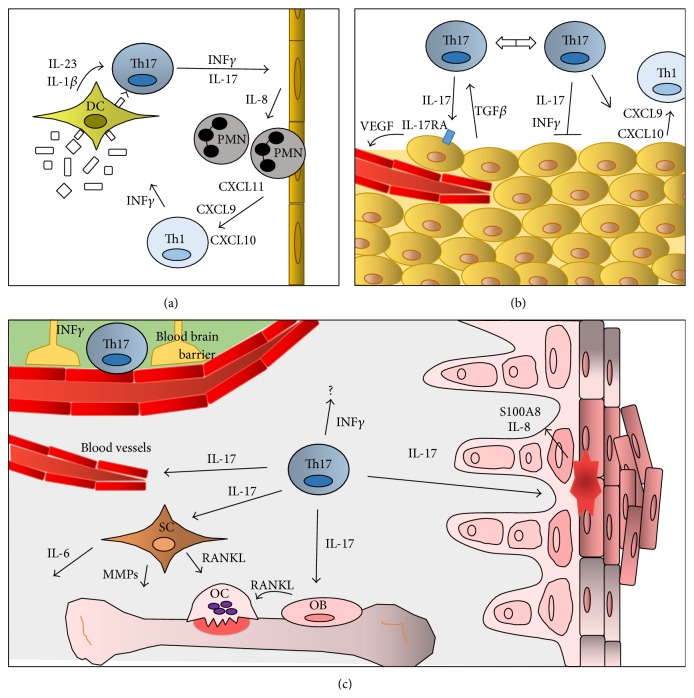
The heterogeneity of Th17 cell phenotype and functions. (a) In immunity to pathogens, (b) in tumour pathology, and (c) in promotion of autoimmune diseases. (a) Dendritic cells (DCs) are stimulated by pathogens to present antigens to induce the differentiation of naïve T-cells to Th17 cells. DCs produce IL-23 and IL-1*β* that facilitate the differentiation to and expansion of activated Th17 cells. Pathogen-specific Th17 cells produce IL-17 and, in some cases, IFN*γ*. IL-17 production induces IL-8 production by endothelial cells leading to neutrophil recruitment and pathogen eradication. Th17 cells can also recruit effector T cells for pathogen eradication. (b) Tumour microenvironments influence the phenotype of Th17 cells. TGF*β* induces Th17 cells to produce vascular endothelia growth factor (VEGF) that induces angiogenesis. However, whilst IL-17 can promote tumour development, IFN*γ* produced by Th17 cells suppresses tumour development through recruitment of other immune cells. (c) IL-17 can promote chronic inflammation and autoimmune diseases. For example, Th17 cells infiltrate the blood brain barrier in patients with MS. In addition, IL-17 induces inflammation in dermal cells in patients with psoriasis. Furthermore, IL-17 can induce angiogenesis and the production of other cytokines-, proteases-, and the receptor activator of nuclear factor kappa-B ligand (RANK-L) from synoviocytes (SC) and osteoblasts (OB) in the synovium of RA patients activating osteoclasts (OC).

For certain bacteria, fungi, and at least one virus, vaccination that initiates Th17 responses can, therefore, confer effective immune protection. Such vaccines often induce immunity at mucosal surfaces that are dependent either on a switch from IL-17-producing T cells to IL-17- and IFN*γ*-producing cells, or a Th17-dependent recruitment of IFN*γ* producing Th1 cells (Figure 2(a)). In most situations during recall responses, key cells that are eventually recruited appear to be neutrophils, which facilitate the eradication of pathogens [51, 55, 59, 64]. Issues to consider during development of a vaccine inducing Th17 protective immunity are the role of adjuvants and whether specific augmentation of the Th17 cell phenotype can increase a favourable immune response.

## 4. Harnessing Th17-Mediated Protection from Cancer

The ability to augment the protective potential of Th17 cells by vaccination has actively been considered for treating cancer. Several studies have identified Th17 cells in tumour masses [65–67]. However, the role of Th17 cells in immunity to cancer is somewhat controversial. As Th17 cells are characterised by functional/phenotypic plasticity, they are likely, therefore, to be differentially influenced by the complex nature of tumour microenvironments (Figure 2(b)). Gastrointestinal tumour development is, for example, driven by an inflammatory environment due to chronic disease and disrupted barriers or by the bacterial flora [68–70]. Th17 cells present in these malignancies have been shown to promote tumour development. One study observed that Th17 cells defined by expression of the transcription factor BATF and IL-23 receptor were more prevalent in the lamina propria of patients with colitis-associated colon cancer than in healthy individuals [69]. Studies in animal models revealed that Th17 cells were induced to proliferate by IL-23 from adjacent antigen presenting cells (APCs) [69]. Perhaps consistent with the stem cell-like genotype of Th17 cells, *β*-catenin produced by Th17 cells in the colon gradually increases comparing patients with ulcerative colitis and colon cancer [43]. In an animal model, colitis-induced cancer was strongly linked to stabilization of *β*-catenin in T cells. *β*-catenin's binding partner Tcf1 was shown bound to the promoter of ROR*γ*t, thus, leading to increased Th17 cell proliferation and cancer development [43]. The proposition that microorganisms influence the inflammatory tumour microenvironment through stimulating Th17 cells is supported by the outcome of three studies. One study revealed that, early during the development of colorectal cancer, impaired mucus production leads to the loss of barrier functions [68]. This resulted in microbial products accessing tumour sites and leading to the production of IL-23 by myeloid cells which, in turn, increased size of the tumour through inducing Th17 cells [68]. The second study observed that the bacterium enterotoxigenic* Bacteroides fragilis* in the colon caused inflammation and proliferation of colonic epithelia leading to the recruitment of Th17 cells. The resulting combination of hyperproliferation of the epithelia, Th17 recruitment, and expansion led to chronic inflammation and colon carcinogenesis [70]. The third study observed that released enteropathogenic bacteria-secreted particles stimulated the intestinal epithelium to produce exosome-like nanoparticles that promoted colon cancer. The nanoparticles, intestinal mucosa-derived exosome-like nanoparticles, contained sphingosine-1-phosphate, CCL20, and PGE_2_. CCL20 recruited T cells from circulation while PGE_2_ facilitated Th17 cell differentiation leading to the development of colon cancer [27]. Although IL-17 can mediate tumours through causing or enhancing chronic inflammation, the cytokine can also act directly on tumour cells. One study reported that the IL-17 receptor A (IL-17RA) was expressed on transformed epithelial cells and these developed into colorectal tumours. These epithelial cells where the main site for protumorigenic activity by IL-17 (Figure 2(b)) [71]. Also the inflammatory environment at other sites than the gastrointestinal tract can induce tumour promoting Th17 cells. Two studies showed that the inflammatory environments in the skin induced by tumour cells or by tumour-derived fibroblasts led to such Th17 recruitment and IL-17-dependent tumour development [72, 73].

Some studies have revealed that Th17 cells can promote cancer if they are the only immune cells found in the tumour but have protective functions in the presence of other immune cells [74]. CD39 and CD73 ectonucleotidase-expressing Th17 cells, induced by IL-6 and TGF-*β*, were reported to be immunosuppressive in several mouse tumours [75]. The ectonucleotidases-degraded ATP led to adenosine release and, subsequently, suppression of helper CD4^+^ and cytotoxic CD8^+^ T cell effector functions. TGF*β* which is commonly found in tumour microenvironments has, therefore, been suggested to augment the immune suppressive and tumour-promoting functions of Th17 cells [75, 76]. Th17 cells have also been reported to promote cancer by virtue of their ability to induce angiogenesis [65]. Zhang and colleagues noted that increased numbers of intratumoral IL-17-producing cells correlated with microvessel density in the tumours and with poor survival of patients with hepatocellular carcinoma [67]. Another study reported that high IL-17 levels in patients with colorectal carcinoma correlated with bad prognosis. In these tumours, Th17 cells facilitate angiogenesis through their ability to induce the production of vascular endothelial growth factor (VEGF) from cancer cells [77]. Indeed, Wang and colleagues reported on the dependence of angiogenesis and experimental melanoma tumour growth on IL-17 [78]. Interestingly, however, angiogenesis promoted by IL-17 was reduced considerably when IFN*γ* ^+^ cells were present in the tumour. This observation could be taken as support for the notion that the presence of other immune cells within tumours promotes a protective role by Th17 cells [78]. The notion that coexpression of IFN*γ* and IL-17 is favourable for tumour immunity was supported by another study of ovarian cancers [66]. In this study, the importance of Th17 cells was suggested by the low number of tumour-infiltrating Th17 cells and the low level of IL-17 in the ascites in patients with more advanced disease. Through the synergistic action between IL-17 and IFN*γ*, Th17 cells induced the production of CXCL9 and CXCL10 to recruit effector T cells [66]. Muranski and colleagues also showed that adoptive transfer of Th17 cells led to the killing of established tumours more efficiently than the transfer of other effector T cell subsets [18]. The authors noted that IL-17^+^IFN*γ* ^+^ T cells were more efficient in cancer eradication than IFN*γ* ^+^ T cells. This was believed to be because the cells were not terminally differentiated and less prone to apoptosis than terminally differentiated IFN*γ* ^+^ Th1 cells [18]. The potential involvement of IFN*γ* ^+^ Th17 cells in providing protective immunity to cancer was highlighted in two further studies. In an animal model involving transplanted solid tumour, bacterial DNA (CpG-) stimulated plasmacytoid DCs (pDCs) presented tumour antigens to Th17 cells. The ability to shrink tumours was dependent on this antigen presentation to Th17 cells and the cells also gaining the ability to produce IFN*γ* [48]. Immune cells including cytotoxic CD8^+^ T cells were subsequently recruited by Th17 cells, mediating protective antitumour immunity [48]. The inducible costimulatory receptor (ICOS) is expressed on Th17 cells and other immune cells. Interestingly, stimulation of ICOS on Th17 cells* in vitro* induced IFN*γ* production (Figure 1). In contrast, anti-CD28 costimulation favoured an IL-17 phenotype only [79] Furthermore, IFN*γ* ^+^ Th17 cells developed through costimulation with ICOS ligand were superior to cells costimulated with anti-CD28 in regression of human tumour engrafted into mice [79].

In conclusion, it is evident that Th17 cells can have paradoxical roles in cancer (Figure 2). It is not currently established if it would be possible to pharmacologically modulate Th17 cells for suppressing cancer. Inhibition of Th17 cells by anti-IL17 therapy or through inhibition of ROR*γ*t could be favourable in situations where Th17 have angiogenic activity [80–83]. Although anti-IL17 therapy has been shown to worsen Crohn's disease, a study using an animal model of colonic tumour revealed that the therapy can be efficacious as a neoadjuvant in combination with chemotherapy when gastric inflammation is localized to the tumour area [71]. In settings where the tumour microenvironment alters Th17 function to one that augments tumour development, therapeutic strategies could be considered to harness the cells to a protective phenotype such as inducing the cells to coproduce IFN*γ*. This suggestion is based on observations cited above that protective immunity to tumours by Th17-like cells is more likely to be achieved if the tumour contains T cells that produce IFN*γ* [74]. Specific stimulation of the coreceptor ICOS was reported to be capable of skewing Th17 cells to become IFN*γ*/IL-17-producing cells which have been revealed to be beneficial for tumour suppression [18, 79]. Furthermore, vaccination with tumour antigens using peptidoglycan or CpG as adjuvants for the activation of DCs or pDCs, respectively, has also been shown to augment a favourable Th17 response [47, 48]. Based on the observations, novel pharmacological agents and adjuvants that can alter the phenotype of Th17 cells should be tested in animal models of cancer. ICOS engagement has, however, also been associated with promotion of autoimmune diseases as its stimulation increases the number of effector Th17 cells [84]. Each agent developed for the purpose of inducing cancer-suppressing Th17 cells should, therefore, be tested both for antitumour properties and the ability to promote autoimmunity.

## 5. Therapeutic Considerations Involving Th17 Cells in Autoimmune Diseases

In addition to their critical role in providing effective immunity to pathogens, especially on mucocutaneous membranes and possible involvement in tumour eradication, Th17 cells have been implicated in autoimmune diseases [19–23, 85]. For example, there is good evidence for multiple roles for Th17 cells and for IL-17 in plaque growth in patients with psoriasis, synovial inflammation, increased angiogenesis and bone degradation in patient with RA and ankylosing spondylitis. The cells have also been suggested to have a role in disruption of the blood brain barrier in multiple sclerosis (Figure 2(c)) [19, 20, 85]. The beneficial therapeutic effect of the anti-IL-17 antibody ixekizumab and the anti-IL-17 receptor antibody brodalumab in psoriasis, psoriatic arthritis, ankylosing spondylitis, and refractory RA is further evidence for the involvement of Th17 cells in these disorders [19–23]. Novel strategies are under development for selective targeting of Th17 cells through inhibiting the nuclear receptor ROR*γ*t [80–83].

As cited above, the available evidence suggests that IFN*γ*-producing Th17 cells are beneficial when considering the development of vaccines for infectious diseases and in some cancers [18, 48, 51, 79]. However, several studies have implicated IFN*γ* ^+^ Th17 cells in the pathogenicity of autoimmune diseases. For example, experimental colitis in mice is driven by IL-23-induced IFN*γ* ^+^IL17^+^ T cells that express the IL-23 receptor [86]. Importantly, the same cells were found in the lamina propria of the intestine in patients with Crohn's disease [86, 87]. In juvenile arthritis, Th17 cells change to a Th1 phenotype while migrating from the circulation to inflamed joints. This conversion can be facilitated* in vitro* by incubating Th17 cells with TGF*β* and high IL-12 levels [88]. Highly enriched Th17 cells from diabetic BDC2.5 transgenic mice adoptively transferred diabetes to NOD/SCID recipients, conferred pathology when the cells were modified to a Th1 phenotype [89]. Furthermore, circulating Th17 cells from relapsing MS patients show an increased propensity to change to IFN*γ*-producing Th17 cells. These cells were also found in brain tissues of patients with multiple sclerosis (MS) [90]. In the animal model of MS, experimental allergic encephalomyelitis (EAE), IFN*γ*-producing Th17 cells preferentially crossed the blood brain barrier to accumulate in the central nervous system. The IL-17^+^IFN*γ* ^+^ T cells displayed better migratory potential than IFN*γ*-producing Th1 cells or non-IFN*γ*-producing Th17 cells [90]. However, a number of other studies disagree with the notion that IFN*γ*-producing Th17 cells are pathogenic in autoimmune diseases. For example, in mice with EAE neither IL-17 nor IFN*γ* deficiency inhibited disease development and symptoms [91, 92]. Instead, it was noted that Th17 cells conferred pathology when the cells gained the ability to produce GM-CSF [93, 94]. Furthermore, two recent studies revealed that, in EAE and in patients with MS, GM-CSF is produced by a separate T cell subset than by Th17 cells [95, 96]. In addition, overexpression of T-bet in T cells during collagen induced arthritis (CIA) was found to reduce bone erosion [97]. Studies in our laboratories have shown that patients with RA who do not respond to treatment with biologic anti-TNF*α* agents produce high levels of IL-17 [98]. Although IL-17 induces bone resorption in patients with RA, IFN*γ*, perhaps paradoxically, is associated with less bone erosion indicating that the development of IL-17^+^IFN*γ* ^+^ phenotype could associate with a better outcome at least for bone loss in the disease [99]. Taken together, these observations suggest that although Th17 cells have pathogenic roles in autoimmune diseases there is also evidence to suggest that not all Th17 cell subsets confer pathology. Furthermore, as cited above, the presence of IFN*γ*-producing Th17 cells could have beneficial protective roles in infections and cancer [18, 51]. There is also evidence that some Th17 cells in patients with RA treated with biologic anti-TNF*α* agents gained a functionally distinct phenotype and produced IL-10 [37]. Clearly, therefore, there is more to discover regarding how Th17 cells and how their plasticity contribute to protective immunity and to inflammatory diseases.

Although augmenting Th17-mediated immunity by vaccination holds promise for infectious diseases and, possibly, for cancer, one caveat is that the approach will not be without risks of promoting autoimmune inflammatory diseases. It is, therefore, of great importance to determine whether different antigens have different propensities to induce Th17-mediated autoimmunity and whether some individuals are more predisposed genetically to such responses [45, 100]. ICOS engagement might be one such example as activation of pathway associated with the coreceptor has been shown to induce Th17 cells capable of ameliorating cancer but also Th1 and Th17 cells with ability to induce arthritis [79, 84]. Whether or not induced Th17 cells could mediate or trigger autoimmune diseases in susceptible individuals should be evaluated in experimental models of diseases such in CIA and in EAE. So far, however, no study has reported on the development of autoimmune symptoms through inducing Th17-mediated responses to infectious pathogens. In humans, a polymorphism in the* DECTIN1* gene, leading to reduced ability to stimulate IL-17 production, was not associated with susceptibility to or severity of RA [101]. It is also possible that the Th17 subset(s) that is protective in infectious diseases could be distinct from Th17 cells that promote autoimmune disease pathology as has been suggested [25]. Indeed, there is some evidence to show that patients with RA have Th17 cells that are unable to eradicate* C. albicans* suggesting that the role of these cells in pathology may not directly be associated with their ability to produce IL-17 [102]. In this respect, IL-17 is known to confer protection of inflamed gastrointestinal tracts from fungal infections suggesting that future treatment options could benefit from avoiding simple blockade of the cytokine [24]. If manipulation of Th17 is safe, it would be of interest to know whether augmenting the transcription factor RUNX1 for IL-17^+^IFN*γ* ^+^ T cell phenotype induction will be beneficial in treating patients with cancer and autoimmune diseases.

## 6. Concluding Remarks

There is a steady increase in knowledge about Th17 cells and their role in different clinical settings since they were first discovered in 2006. As these cells are involved both in immunity against pathogens and in promoting autoimmune diseases and perhaps some cancers, the development of therapeutic strategies to harness Th17-mediated responses is already an area of huge interest. Augmentation of Th17 cell-dependent but antibody-independent vaccination for certain pathogens is under active development. Such an approach would have the benefit of triggering protective immunity that is serotype-independent and suitable for children, immune compromised individuals, and individuals with impaired B cell-mediated immunity. However, the fact that at least some Th17 subsets could play pathogenic roles in some autoimmune diseases and, possibly, in some cancers, targeted inhibition or modulation of the function of these cells could also be immensely beneficial. Strategies that rely on developing novel specific molecules to modulate Th17 cells are already underway. Perhaps what complicates the development of such strategies is the plasticity of the phenotype and functions of Th17 cells which are, clearly, noted to be more than other T cell subsets. This plasticity is likely to hamper the development of strategies to modulate Th17 cells in cancer as the tumour microenvironment and presence of other immune cells could influence the outcome of such treatments. Specific augmentation, for example, by inducing IFN*γ* production by Th17 cells, has been shown to be beneficial in many infectious diseases and in some cancers. Although IFN*γ*-producing Th17 cells have been suggested to be associated with autoimmune diseases in some studies, there is currently limited evidence that such cells can actually lead to autoimmune disease development. Future treatment options for vaccination for infectious diseases, cancers, and autoimmune diseases might involve augmentation of distinct Th17 cell subsets for beneficial outcomes. Th17 cells are arguably the T cell subset that has coevolved with us for the longest time. Increased knowledge of these cells in health and in disease could be of immense future benefit.
